# Early acute pancreatitis in a child with compound heterozygosis ∆F508/R1438W/Y1032C cystic fibrosis: a case report

**DOI:** 10.1186/1752-1947-7-188

**Published:** 2013-07-24

**Authors:** Salvatore Leonardi, Andrea Domenico Praticò, Novella Rotolo, Giovanna Di Dio, Elena Lionetti, Mario La Rosa

**Affiliations:** 1 Department of Medical and Pediatric Science, Unit of Broncho-Pneumology and Cystic Fibrosis, University of Catania, Via Santa Sofia 78, Catania 95123, Italy

**Keywords:** Pancreatitis, Cystic fibrosis, CFTR, ∆F508-R1438W and Y1032C, Mild phenotype

## Abstract

**Introduction:**

Recent studies suggest an important role of the cystic fibrosis transmembrane conductance regulator gene in the development of pancreatitis. It occurs approximately in 20% of patients with cystic fibrosis and almost exclusively in pancreatic sufficient people. Newborn screening and improved panels of deoxyribonucleic acid mutation analysis techniques are revealing more rare and nonclassical pictures of the disease, generally associated with pancreatic sufficiency and with an increased risk of developing pancreatitis. Mutations R1438 and Y1032 are considered rare mutations, and, when singularly associated with ∆F508, lead to a mild phenotype with pancreatic sufficiency and no detectable respiratory involvement.

**Case presentation:**

We present the case of a Caucasian girl, aged six years, whose genotype was characterized by three different mutations ∆F508, R1438W and Y1032C, never reported, together, in the same patient. She presented with a positive immunoreactive trypsinogen screening, a borderline sweat test, and, in the first years, a favorable pulmonary course, and pancreatic sufficiency. At the age of six years, she presented with a sudden episode of acute abdominal pain, anorexia and fever. A diagnosis of pancreatitis was made after clinical and laboratory examinations. Venous rehydration, bowel rest and therapy with ursodeoxycholic acid resulted in complete remission.

The treatment was successful, with normalization of her symptoms and laboratory parameters within four weeks.

**Conclusion:**

There has been a vast expansion in the understanding of the wide range of phenotypes associated with cystic fibrosis transmembrane conductance regulator dysfunction since the discovery of the cystic fibrosis transmembrane conductance regulator gene. The genotype-phenotype correlation in pancreatitis is rare compared to other organ manifestations, since this is seen almost exclusively among pancreatic sufficient patients with cystic fibrosis. Our study supports that compound heterozygosis ∆F508-R1438W/Y1032C is a 'cystic fibrosis-causing genotype' characterized by an immunoreactive trypsinogen positive screening, abnormal sweat chloride testing, and pancreatic sufficiency, with an increased risk of acute pancreatitis at an early age.

## Introduction

Cystic fibrosis (CF) is the most common, potentially lethal, autosomal recessive disorder in western countries (1:2500 live births). The disease is caused by mutations in the gene that encodes for the cystic fibrosis transmembrane conductance regulator (CFTR) protein [1].

The exocrine pancreas is the most reliable phenotypic barometer of CFTR function among all the organs affected [2]. The relationship between the severity of the CFTR genotype and the risk of pancreatitis has been well established and it occurs almost exclusively in patients with CF presenting with mild mutations and pancreatic sufficient patients with CF, and not in patients with pancreatic insufficiency (PI) [2].

Recently, deoxyribonucleic acid (DNA) mutation analysis is revealing new CFTR gene haplotypes but sample size limitations make genotype-phenotype correlation studies difficult [3,4].

To date, the presence of three different mutations in the same patient is rarely reported and it represents a new field of research on genotype-phenotype profiling. We report a case of a seven-year-old girl, affected by cystic fibrosis disease, who presented with acute pancreatitis at six years of age.

## Case presentation

The patient, a Caucasian six-year-old girl, was born at term by nonconsanguineous, healthy parents, with a birth weight of 3.160kg. Her newborn screening test results for cystic fibrosis, performed by immunoreactive trypsinogen (IRT) measurement, were positive on the third and fifteenth day. Her sweat test showed borderline results of chlorine 44mEq/L and 48mEq/L (normal value (n.v.) <40mEq/L). The genetic analysis of the CFTR gene showed compound heterozygosis of ∆F508-R1438W *in cis* and Y032C *in trans* mutations (Table 1). In the first year's growth, her respiratory system and respiratory functions were normal. In fact, at the age of six years, her weight was 20kg (35^th^ percentile for age), her body mass index (BMI) was 14.4 (24^th^ percentile for age); at spirometry, her forced vital capacity (FVC) was 90% and forced expiratory volume (FEV)1 92%, and she had always presented with a low amount of fecal elastase-1.

**Table 1:** Genotype of the patient

	**Cystic fibrosis transmembrane conductance regulator gene haplotype**		
**Mutation**	**Patient**	**Father**	**Mother**
**∆F508**	∆F508/+	+/+	∆F508/+
**R1438W**	R1438W /+	+/+	R1438W/+
**Y1032C**	+/Y1032C	+/Y1032C	+/+

**+** denotes the presence of the wild-type allele.

At six years old, the patient was treated for acute abdominal pain, anorexia and fever. She presented with abdominal pain radiating to the back, mild muscular guarding, decreased bowel sounds and mild scleral icterus. Her laboratory examination results showed an erythrocyte sedimentation rate (ESR) of 51mm/h, increased values of total bilirubin (2.67mg/dL) and direct bilirubin 1.80mg/dL, pancreatic amylase (305U/L, n.v. 0 to 46U/L) and total amylases (465U/L, n.v. 28 to 100U/L) and lipase (310U/L, n.v. 0 to 60U/L). An ultrasound evaluation of the abdomen showed parenchymal hyperechogenity of the pancreas with no images of biliary calculi, or hepatic alterations. Venous rehydration, bowel rest and therapy with ursodeoxycholic acid were successfully performed, with normalization of her symptoms and laboratory parameters within four weeks.

At present, general conditions are good. Her respiratory function is normal on spirometric evaluation, and she is not presenting with bacterial colonization in the respiratory tract. Pancreatic and digestive functions are good: her weight is on the 25^th^ percentile, her height is on the 75^th^ percentile, her pancreatic amylase value and total amylases are within the normal range.

## Discussion

Exocrine pancreas function is a reliable predictor of overall CFTR function and disease severity. Most patients with CF carrying functionally severe mutations on both alleles have a PI phenotype [2], while patients who carry a mild mutation on at least one allele usually have sufficient exocrine pancreatic function. However, while pancreatic insufficiency is a recognized complication of CF, symptomatic pancreatitis represents a rare manifestation of CF, affecting <2% of patients. Specific CFTR genotypes are significantly associated with pancreatitis and paradoxically genotypes associated with otherwise mild phenotypic effects have a greater risk [3]. Clinical presentation of acute pancreatitis in patients with CF is substantially similar to what happens in unaffected people. Together with the 'common' risk factors (alcohol, gallstones, hypercalcemia, hyperlipidemia, malnutrition, abdominal trauma, drugs, infections and radiation), pancreatic sufficient patients with CF present an impaired secretion of pancreatic enzymes due to obstructions in the ducts caused by altered chlorine transportation.

Rosendahl *et al*. [5] investigated 660 patients affected by chronic pancreatitis (CP) and found that combined CF-causing variants increased CP risk 3.4-fold (*P*<0.001), while non-CF-causing variants displayed a 1.5-fold overrepresentation in patients (*P*=0.14). Trans-heterozygosity increased CP risk, with an odds ratio (OR) of 38.7, with 43 out of 660 (6.5%) patients and three out of 1667 (0.2%) controls being trans-heterozygous (*P*<0.0001). They stated that compound and trans-heterozygosity is an overt risk factor for CP.

Our patient presented with a compound heterozygote genotype, characterized by ∆F508/R1438W and Y1032C mutations. It is not uncommon to find, in southern regions of Italy, different combinations of mutations in the same patients. Contrary to what happens in Northern Europe, Italian ancestry varies from Caucasian to Arabic and Middle Eastern population, and the different mutations found are probably due to the higher allelic heterogeneity of the CFTR gene in Italian population. ∆F508 mutation, for instance, is found (in homozygosis or in heterozygosis) in 50% of Italian patients with CF, while in Northern Europe the percentage is higher (70%) [1].

∆F508 has been the first recognized mutation of the CFTR gene, its prevalence in Caucasian population is estimated at 2.8% and in homozygosis it is responsible for more than 70% of the typical form of cystic fibrosis [1].

In our patient, in the same chromosome carrying the ∆F508 mutation, a substitution of a thymine with a cytosine in position 4444 of exon 24 (R1438W mutation) was present. This mutation was first described by Seia *et al*. in a man, 18 years of age, diagnosed with CF at one year due to respiratory symptoms. His sweat test was abnormal (78mmol/L chloride), but he was pancreatic sufficient and his growth and lung function results were normal [6]. In Seia’s report, the S977F variant was identified on the same chromosome, while ∆F508 was identified on the other chromosome (compound heterozygosis R1438W-S977F/∆F508).

Similar to our patient, the association of ∆F508-R1438W *'in cis'* (together with E588V mutation in the other allele) has been already described [7]. This genotype causes a positive IRT newborn screening and a mild presentation of CF with pancreatic sufficiency and no pulmonary disease.

In our patient, we found the rare Y1032C mutation in the other chromosome. This mutation was first identified in a German patient affected by congenital bilateral absence of vas deferens (CBAVD), who presented with a compound heterozygosis Y1032C/∆F508 [8]. The patient presented with recurrent bronchitis and pancreatic sufficiency. Ratbi *et al.*[9] also reported a CBAVD in another patient affected by a compound heterozygosis Y1032C/D1152H mutation.

Both Ren *et al.*[10] and Seia *et al.*[11] included ∆F508/Y032C among the mutations causing a 'milder' form of CF with no clinical symptoms, borderline sweat test, pancreatic sufficiency and no pulmonary disease.

How the alteration in the CFTR protein caused by R1438W and Y1032C may lead to a specific 'pancreatitis-causing' phenotype by the combination with a severe mutation like ∆F508 is unknown, but to date the combination of these two rarely reported mutations with severe mutations has been never associated with acute pancreatitis. It is consistent to think that the combination of the mild deficit caused by these two mild mutations (together with ∆F508) could have caused a temporary obstruction of pancreatic ducts but not a complete pancreatic insufficiency.

## Conclusions

The apparent status of wellness of patients with CF with mild mutations and genotype could mislead the caregivers and let them ignore the possible onset of acute pancreatitis, more common in pancreatic sufficient patients. Our study would support that compound heterozygosis ∆F508-R1438W/Y1032C is a 'CF-causing mutation' characterized by a positive IRT screening, borderline sweat test, and pancreatic sufficiency, with a risk of acute pancreatitis at an early age.

## Consent

Written informed consent was obtained from the patient's next-of-kin for publication of this manuscript and any accompanying images. A copy of the written consent is available for review by the Editor-in-Chief of this journal.

## Competing interests

The authors declare that they have no competing interests.

## Authors’ contributions

SL and ADP analyzed and interpreted the patient data regarding the mutations and performed the review of the literature. NR, GDD and EL performed the clinical examination of the patients, and were major contributors in writing the manuscript. MLR revised the manuscript. All authors read and approved the final manuscript.
